# A Comparison of the Imaging Performance of High Resolution Ultrasound Scanners for Preclinical Imaging

**DOI:** 10.1016/j.ultrasmedbio.2010.11.010

**Published:** 2011-03

**Authors:** Carmel M. Moran, Stephen D. Pye, William Ellis, Anna Janeczko, Keith D. Morris, Alan S. McNeilly, Hamish M. Fraser

**Affiliations:** ∗ Medical Physics, Centre for Cardiovascular Sciences, The Queens Medical Research Institute, University of Edinburgh, Edinburgh, United Kingdom; † Medical Physics Department, NHS Lothian, Royal Infirmary of Edinburgh, Edinburgh, United Kingdom; ‡ MRC Human Reproductive Sciences Unit, Centre for Reproductive Biology, University of Edinburgh, Edinburgh, United Kingdom

**Keywords:** High resolution ultrasound, Edinburgh Pipe Phantom, Preclinical, Resolution integral

## Abstract

Nine ultrasound transducers from six ultrasound scanners were assessed for their utility for preclinical ultrasound imaging. The transducers were: L8-16, L10-22 (Diasus; Dynamic Imaging Ltd., Livingston, UK); L17-5, L15-7io (iU22; Philips, Seattle, WA, USA), HFL38/13-6 (MicroMaxx; Sonosite Inc., Bothell, WA, USA); il3Lv (Vivid 5; GE, Fairfield, CT, USA), RMV 704 (Vevo 770; Visualsonics Inc., Toronto, Canada) and MS550S, MS550D (Vevo 2100; Visualsonics Inc.). A quantitative analysis of the ultrasound images from all nine transducers employed measurements of the *resolution integral* as an indication of the versatility and technology of the ultrasound scanners. Two other parameters derived from the resolution integral, the *characteristic resolution* and *depth of field*, were used to characterise imaging performance. Six of these transducers were also assessed qualitatively by ultrasonically scanning 59 female common marmosets (*Callithrix jacchus)* yielding a total of 215 scans. The quantitative measurements for each of the transducers were consistent with the results obtained in the qualitative *in vivo* assessment. Over a 0–10 mm imaging depth, the values of the resolution integral, characteristic resolution and depth of field, measured using the Edinburgh Pipe Phantom, ranged in magnitude from 7–72, 93–930 μm and 3.3–9.2 mm respectively. The largest resolution integrals were obtained using the Vevo 770 and Vevo 2100 scanners. The Edinburgh Pipe Phantom provides a quantitative method of characterising the imaging performance of preclinical imaging scanners. (E-mail: Carmel.Moran@ed.ac.uk)

## Introduction and Literature

Research using small animals has played and continues to play a key role in the advancement of biological, biomedical and veterinary science. In particular, the mouse model has become increasingly popular as a research model due to the fact that the human and mouse genomes are remarkably similar with over 85% of the genomic sequences in mice identical to those found in humans (National Human Genome Research Institute, http://www.genome.gov). This knowledge of the genomic sequence enables researchers to knock-out specific genes enabling the creation of many genetically engineered mouse models of human diseases and, consequently, helping to decipher disease mechanisms. An additional advantage of using rat and mouse models is their relatively low maintenance costs and large litter-sizes. The requirement to monitor disease progression or to determine which mice are exhibiting a specific genotype may require the sacrifice of many animals. A non-invasive imaging technique that is capable of high throughput, relatively inexpensive and with sufficient resolution to quantify the parameters of interest with high accuracy may reduce the number of animals that are sacrificed. A range of imaging techniques are available using knowledge and technology gained from their clinical counterparts. These include computed tomography (CT), magnetic resonance imaging (MRI), positron emission tomography (PET), optical coherence tomography (OCT) and high frequency ultrasound imaging. In the clinical setting, it has been shown that cardiac imaging with ultrasound provides the most cost-effective imaging modality (Picano 2005) whilst Olive and Tuveson (2006) have demonstrated, with respect to preclinical tumour imaging, that ultrasound demonstrates high resolution with a low capital cost. Moreover, the advent of a commercial ultrasound scanner designed specifically for *in vivo* microimaging of mice (Foster et al. 2002) has demonstrated the versatility of the technique including the use of ultrasound to assess both embryonic development (Mu et al. 2006; Phoon et al. 2006, Floch’h et al. 2004), reproductive system (Akirav et al. 2005), eye development (Cheung et al. 2007), cardiovascular (Springer et al. 2005, Kaufmann et al. 2007; Zimmerman et al. 2006, Williams et al. 2007), prostate imaging (Wirtzfeld et al. 2005) and cancer therapeutics (Olive and Tuveson 2006; Cheung et al. 2007).

We have previously developed a test object, the Edinburgh Pipe Phantom, to quantitatively characterise ultrasound scanners for clinical applications (MacGillivray et al. 2010). The aim of this article is to describe the development of the Edinburgh Pipe Phantom as a means to assess a range of both clinical and preclinical scanners for preclinical imaging. These results are then discussed in the context of images acquired from a preclinical imaging model (common marmoset [*Callithrix jacchus*]) using six of the transducers previously assessed with the Edinburgh Pipe Phantom.

### Resolution integral

**Fig. 1:**
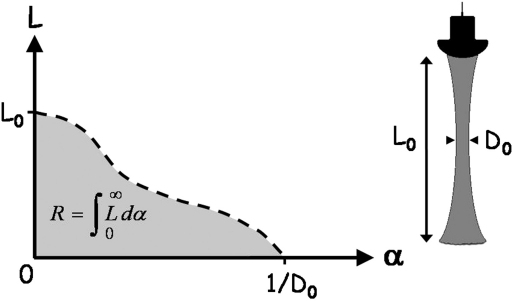
Plot of L against α for a weakly focused ultrasound beam with low contrast penetration L_0_ and a minimum beam width D_0_. α is the reciprocal of beam width, and L(α′) corresponds to the depth range over which the beam width is less than 1/α′. The resolution integral R is equal to the area under the curve.

**Fig. 2:**
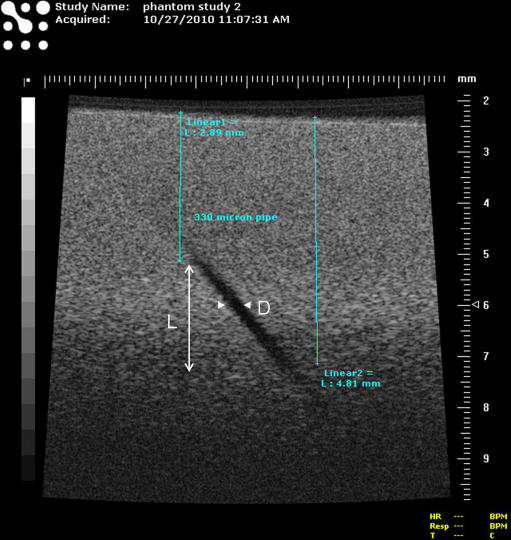
Image of 330 micron pipe using the RMV 704 probe illustrating the measurement of L, the depth range over which the pipe could be imaged. The image of the pipe disappears (*i.e*., the pipe is not visible) when the beam width exceeds the effective diameter of the pipe (shown as D on the image, and defined in the text).

**Fig. 3:**
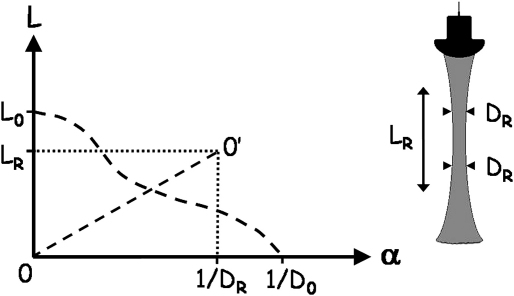
Plot of L against α for the same ultrasound beam illustrated in Figure 1, illustrating depth of field L_R_ and characteristic resolution D_R_. The area under the dashed curve 0-L_0_-1/D_0_ is equal to the resolution integral R. The rectangle 0-L_R_-0’-1/D_R_ also has an area equal to R, and line 00’ bisects both the rectangle and the area under the dashed curve giving R = L_R_/ D_R_.

The quantitative analysis of the ultrasound transducers used in this study employed measurements of the resolution integral made using two Edinburgh Pipe Phantoms. These were composed of tissue-mimicking material (TMM) (Ramnarine et al. 2001) in which were embedded a series of anechoic wall-less pipes of varying diameters (Pye and Ellis 2004). The resolution integral R is essentially the ratio of the penetration of an ultrasound beam to the ultrasound beam width. Highly sensitive transducers combine deep penetration with high resolution, and so the ratio penetration/beam-width is large. Figure 1 illustrates this concept for a weakly focused imaging beam. The abscissa, α, is the inverse of the effective pipe diameter, calculated as the geometric mean diameter of the pipe imaged in the lateral and elevation planes. The ordinate, L, indicates the depth range over which each pipe can be imaged and is the difference between the maximum and minimum depths over which each pipe could be imaged (Fig. 2). Larger pipes are imaged over greater depths than smaller ones, with the low contrast penetration defining the limit of detection for large pipes as α tends to zero. Two other parameters are defined in Figure 3: depth of field L_R_ and characteristic resolution D_R,_ and these are related by R = L_R_/D_R_. The depth of field defines a region of optimum resolution, and the characteristic resolution is representative of the lateral and azimuthal resolution within the depth of field. A transducer suited to a particular imaging application will have a small characteristic resolution together with a sufficiently large depth of field for the specific application. These concepts have been described with reference to clinical scanners (Pye et al. 2004, 2010; MacGillivray et al. 2010).

## Materials and Methods

### Phantom studies

The details of construction of the two Edinburgh Pipe Phantoms can be found elsewhere (Pye et al. 2004; Moran et al. 2008; Moran et al. 2010) and will only be described briefly here. The initial pipe phantom was constructed from a 250 × 250 × 100 mm block of TMM. The TMM had attenuation coefficient of 0.5 dB cm^−1^ MHz^−1^ and speed of sound of 1540 ms^−1^. Eleven wall-less pipes ranging in diameter from 0.35 mm to 7.9 mm were moulded into the TMM at an angle of 40° to the vertical. A second pipe phantom was constructed with a range of pipe diameters from 0.045 mm to 1.47 mm embedded in a series of cylinders of TMM of diameter 60 mm and height of 40 mm. The wall-less pipes were made by inserting stainless steel tube, rod, wire and surgical suture of appropriate diameter into the TMM, at an angle of 40° to the vertical, whilst in its liquid phase. Once the TMM had set, the rods were withdrawn under submersion in a water and glycerol mixture with a speed of sound of 1540 ms^−1^.

**Fig. 4:**
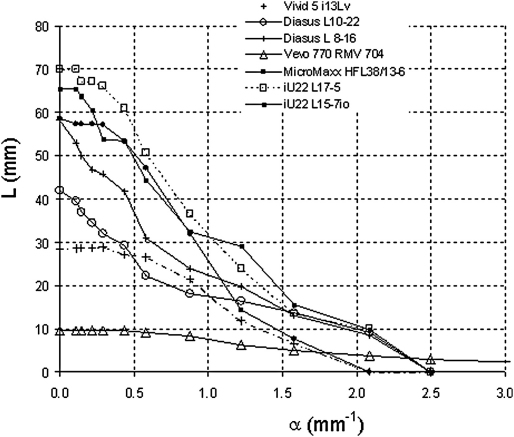
Plot of L (depth over which pipe can be visualized) against α (1/effective pipe diameter) for each of the transducers assessed using the initial pipe phantom.

**Fig. 5:**
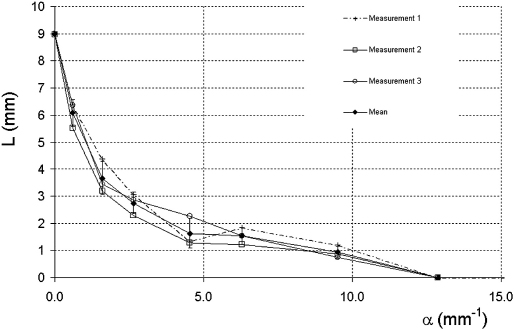
Plot of L against α for the RMV 704 transducer using the second pipe phantom. The measurements were taken on three separate occasions over a 15-month period.

**Fig. 6:**
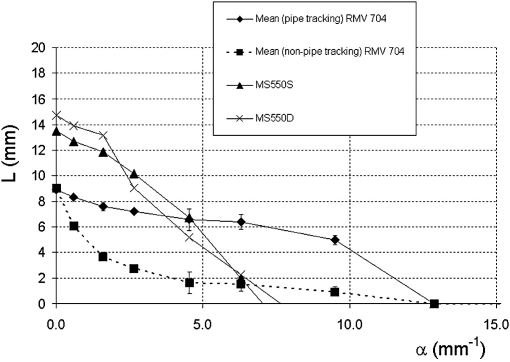
Plot of L against α for Vevo 770 RMV 704 transducer and Vevo 2100 MS550S and MS550D transducers. For the RMV 704 transducer, the curves represent the results from the two methods of measuring the resolution integral (tracking and non-tracking). The values are the mean and standard deviation of three sets of measurements.

Each ultrasound transducer that was assessed was placed on the surface of the TMM and coupled to it with water/glycerol mixture. An image of the largest diameter pipe was obtained and the upper portion of the pipe was imaged with the pipe axis positioned in the scan plane of the transducer. The image was optimized (including adjustment of focal position for array transducers) to visualise the pipe as superficially as possible, with the transducer held vertically, and the most superficial region of the pipe that could be detected positioned directly below the transducer (within ±10° of the vertical). The image was then frozen and a visual assessment was made of the minimum depth at which the pipe could be visualised. To aid in this assessment, a mask of buff-coloured paper with a slot of width approximately 15 times the wavelength of the centre frequency of the transducer was used to mask out echoes from neighbouring regions of the pipe and TMM. The mask was placed onto the frozen ultrasound image and the slot was moved up and down along the length of the pipe image to allow the observer to visualise each short section of the pipe in turn, compare it with adjacent speckle and identify and measure the minimum depth below the scanning surface at which the pipe could be confidently detected. The transducer was then repositioned and the same procedure was carried out to identify the maximum depth below the scanning surface at which the pipe could confidently be detected. The difference between the maximum and minimum depths at which the pipe could be detected is equal to L, the ordinate of the data points in Figures 1, 3, 4, 5 and 6. This procedure was repeated for each pipe and, as previously described, each value of L was plotted against α, the reciprocal of the effective pipe diameter. The effective pipe diameter D shown in Figure 2 is calculated as the geometric mean of the pipe diameter in the image and elevation planes and is equal to d/√(cos 40°) for a pipe of diameter d. Using the initial pipe phantom, up to 11 pipes were scanned. Each pipe measurement corresponds to one data-point on the curves in Figure 4. The low contrast penetration depth of the transducer was determined by measuring the maximum depth at which speckle could be identified distinctly from system noise while imaging in real time. This is shown in Figures 4, 5 and 6 as the intersection of each data-curve with the ordinate.

The authors obtained access to a Vevo 2100 scanner at a later stage, after the *in vivo* study had been completed. This scanner is based on solid-state array technology and two linear array transducers, the MS550S and MS550D, were assessed using the pipe phantoms. These results are included for comparison.

The nine ultrasound transducers tested are listed in Table 1. Only the Vevo 770 RMV 704 (Visualsonics Inc., Toronto, Canada) transducer and the Vevo 2100 MS550S and MS550D were tested using the second pipe phantom. The measurements taken using the RMV 704 transducer were on a different Vevo 770 scanner to that used for the preclinical imaging and were carried out in the Biological Research Facility of the University of Edinburgh, UK.

### *In vivo* studies

The study described in this article was carried out for the MRC Human Reproductive Sciences Unit, which has a programme of research on the manipulation of pituitary-ovarian-uterine function during the normal ovarian cycle and in early pregnancy (Fraser and Duncan 2009; Wulff et al. 2002). All ultrasound examinations were performed in accordance with home office regulations and approved by the local ethics committee in a manner similar to that described by Oerke et al. (1996). A total of 59 adult female marmosets being studied for changes in ovarian follicular development throughout the ovarian cycle were ultrasonically scanned over a 6-month period, yielding a total of 215 scans. The number of scans obtained for each marmoset varied dependent upon the programme of research in which the animals were enrolled (17 marmosets receiving only 1 scan, 39 marmosets receiving between 2 and 10 scans and 2 marmosets receiving 12 and 16 scans, respectively).

All marmosets were imaged using a Diasus (Dynamic Imaging Ltd., Livingston, UK) ultrasound scanner using either an L8-16 transducer or L10-22 transducer. On four separate occasions, marmosets were additionally imaged using one of the transducer/scanner combinations under evaluation. These marmosets were initially scanned using the Diasus and then immediately scanned using the transducer/scanner combination under evaluation. Except for the Vevo 770 scans, all imaging was undertaken by the same sonographer who had considerable experience in ultrasonically scanning marmoset ovaries. For the Vevo 770 scanning session, an experienced Visualsonics demonstrator scanned the animals. Nine marmosets were scanned with the Sonosite MicroMaxx (Sonosite Inc., Bothell, WA, USA) ultrasound scanner, 14 marmosets with the Visualsonics Vevo 770 and 11 marmosets with the Philips (Seattle, WA, USA) iU22 and HD11 scanners. Based on the initial results from our phantom studies, described above, no animals were scanned with the Vivid 5 scanner (GE, Fairfield, CT, USA).

**Fig. 7:**
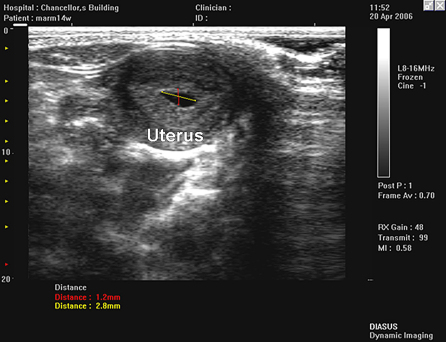
Uterus of early pregnancy in the marmoset, using a Diasus L10-22 transducer.

**Fig. 8:**
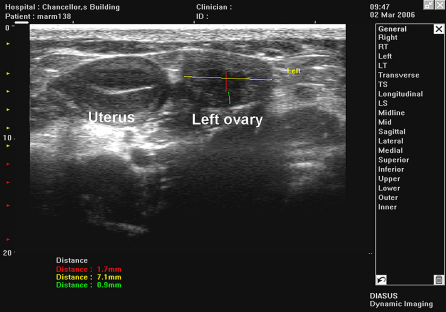
Marmoset uterus and left ovary with dimensions of ovary (7.1 mm) and two follicles (1.7 mm and 0.9 mm) imaged using a Diasus scanner L8-16 transducer.

The marmosets were hand-held by trained animal technicians and were fully conscious throughout the scanning examination. Scans lasted no longer than 3 min. The abdomen of the marmoset was shaved, warmed gel was layered onto the skin and the transducer was placed gently onto the surface of the abdomen. The location of the uterus was identified on the ultrasound scan (Fig. 7). As a general rule, at least one of the ovaries proved to be located in the same field of view as the uterus and was easily located (Fig. 8). The second ovary was often located more cranial to the uterus and on the opposite side. Follicles were identified as circular anechoic structures with distinct boundaries. The marmoset commonly ovulates two or three follicles per cycle and resulting corpora lutea were identified by less distinct boundaries and increased internal echogenicity within a circular structure. Once an ovary was located, the transducer was moved cranially until the top of the ovary was located and then subsequently moved toward the lower abdomen to scan through the whole ovary. The number and size of follicles and corpora lutea within each ovary was determined.

In addition to the 59 animals scanned, an additional seven animals from our breeding colony were scanned during the early stages of pregnancy.

## Results

### Phantom studies

Figures 4, 5 and 6 illustrate the quantitative data obtained from each of the nine transducers using the Edinburgh Pipe Phantoms. The curve for each of the transducers is composed of a minimum of seven data points, each data point corresponding to a specific pipe. The resolution integral R, characteristic resolution D_R_ and depth of field L_R_ were calculated over the full range of depths imaged (Table 2) and also over a depth range of 0–10mm appropriate for preclinical imaging (Table 3).

The resolution integral is equal to the area under each of the curves in Figures 4, 5 and 6. In Figure 4, it is evident that the curve for the Vevo 770 RMV 704 transducer does not form a closed curve bounded by the abscissa, indicating that the smallest wall-less pipe in the initial phantom (diameter 0.35 mm, α = 2.5 mm^−1^) could be resolved over a range of depths. The results of the other transducers tested all formed closed curves. The iU22 transducers and Diasus transducers were able to resolve pipe sizes of diameter 0.42 mm (α = 2.08 mm^−1^) but unable to resolve pipes of diameter 0.35 mm (α = 2.5 mm^−1^) whilst the Vivid 5 and MicroMaxx transducers were unable to resolve the 0.42 mm diameter pipe size.

To make a measurement of resolution integral for the Vevo 770 RMV 704 transducer, it was necessary to use the second pipe phantom containing smaller diameter pipes. The results are shown in Figure 5, where each data point is the mean of three separate measurements on the same phantom, using the same transducer over a period of 15 months. The Vevo 770 RMV 704 probe was able to resolve pipe sizes of diameter 0.092 mm (α = 9.5 mm^−1^) but unable to resolve pipes of diameter 0.068 mm. An alternative approach to measuring the resolution integral for the Vevo 770 scanner was also employed by adjusting the position of the transducer so that the focal region lay at the specific depth of interest. This was achieved by varying the distance of the transducer from the phantom, so that initially the top of each pipe was visualized in the focal region and then the transducer was moved closer to the TMM so that the pipe could be imaged as deep into the TMM as possible. By using this method of “tracking” the visualization of the pipes through the phantom, all pipes resolved by the RMV 704 could be visualized at the surface of the TMM. Distance L was then measured as the vertical distance between the surface of the TMM and the maximum depth at which the pipe could be visualized. This was repeated for each pipe in turn and the mean of three measurements was plotted (Fig. 6). This tracking technique is consistent with the standard operation of the Vevo 770 for preclinical imaging, where coupling gel is often used as a stand-off to ensure that anatomical structures of interest can be positioned in the focal region. The results obtained from the Vevo 2100 MS550D and MS550S are also shown on Figure 6. Both transducers were able to resolve pipe sizes of diameter 0.139 mm (α = 6.3 mm^−1^) but unable to resolve pipes of diameter 0.092 mm.

### *In vivo* studies

Due to the nature of the *in vivo* study, the results presented below are predominantly qualitative in nature.

Ultrasound images acquired using the Visualsonics RMV 704 transducer showed greater detail within the ovarian structures than those obtained using either of the Diasus transducers and, in three ovaries, the Vevo 770 RMV 704 showed a larger number of follicles than were detected with the Diasus L8-16. This finding was later confirmed by histologic analysis.

The scans obtained using the MicroMaxx did not provide any additional qualitative information on the number and location of follicles in comparison to the scans obtained using the Diasus and the imaging was disappointing in the lack of detail that was visualized *in vivo* compared with both the Diasus transducers. Both Philips transducers (L15-7io on the HD11, L17-5 on the iU22) demonstrated as good a differentiation of the number and size of follicles within the ovaries as the Diasus scanner. Based on these *in vivo* results, a qualitative ranking of the transducers most suitable for preclinical scanning applications was established (Table 4).

**Fig. 9:**
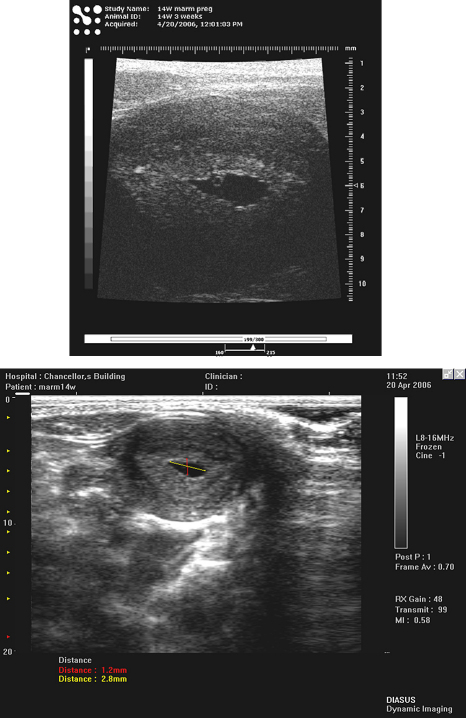
Top: Visualsonics Vevo 770 RMV 704 image of early pregnant uterus in a marmoset showing conceptus and endometrium. Bottom: Image acquired using a Diasus L8-16 probe on the same marmoset. No conceptus or endometrium visible.

Pregnancy was established by the ultrasonic appearance of an anechoic space in the centre of the uterus (Fig. 7). Specifically in two instances where the ultrasonic image did not identify pregnancy, but palpation confirmed pregnancy, the animals miscarried at a later stage. The Diasus scanner had 100% effective determination of pregnancy and could detect pregnancy between 2 and 3 weeks post ovulation, which was subsequently confirmed by manual palpation of the uterus at 6 weeks. In addition, in one early pregnancy imaging session, the Vevo 770 scanner demonstrated the conceptus and endometrium in an early pregnancy, which was not visible in any images acquired with the Diasus L8-16 transducer (Fig. 9).

## Discussion

A transducer that is to be used for preclinical applications requires high resolution combined with sufficient depth of field for the specific application. In preclinical applications, this depth of field is usually, but not always limited, to approximately 10 mm. In Table 2, the transducers are ranked with respect to characteristic resolution such that those with optimal characteristic resolution are placed toward the top of the table. Although both the Vevo 770 RMV 704 (untracked) and the Vevo 2100 preclinical probes are ranked toward the top of Table 2, the corresponding depth of field of these transducers is limited. In comparison, the two iU22 transducers and the MicroMaxx transducer demonstrated the greatest depths of field indicating the versatility of these transducers over a range of depths. The characteristic resolution of the clinical scanners varied significantly from 840 μm for the MicroMaxx to 520 μm for the Diasus L10-22 transducer. These results were consistent with the observations of the *in vivo* studies (Table 4) using the clinical transducers. The iU22 transducers needed little optimization to obtain good quality images from the ovaries but in general showed no more detail than optimized images from the Diasus scanner. (Note, however, that the L15-7io transducer on the Philips scanner was tested on the iU22 scanner for the quantitative study but used to image the marmosets on the HD11). The lack of detail obtained *in vivo* using the MicroMaxx transducer compared with both the Diasus transducers is consistent with its larger characteristic resolution (840 μm vs. 600 and 520 μm).

The Vevo 770 RMV 704 data are of interest. Using the tracking technique, it demonstrates a large resolution integral (R = 72), the smallest characteristic resolution (93 μm) and a depth of field of 6.7 mm. Using the non-tracking technique, the resolution integral and depth of field are substantially smaller (R = 25, L_R_ = 3.3 mm) and the characteristic resolution is larger (132 μm). Unlike the other transducers used in this study, the RMV 704 is based on single element technology with a greater degree of focussing. Consequently, over the limited depth range of the focal region, the transducer performs well giving a small characteristic resolution. When used outside that range, the ability of the transducer to resolve small objects reduces at a much faster rate than the array transducers routinely used in clinical imaging. This was evident from the *in vivo* studies where, dependent on the size of the animal to be scanned, Vevo 770 transducers with differing focal positions were used to obtain optimal images. Indeed, in the Vevo 770 image in Figure 9 of the pregnant uterus, it is evident that the posterior edge of the uterus is not visible due to its distance from the focal region of the transducer.

For preclinical imaging, structures of interest are generally located at less than 10 mm depth and the data presented in Table 3 are particularly relevant. The resolution integral, characteristic resolution and depth of field are calculated over a depth of 1–10 mm and ranked with respect to characteristic resolution (as in Table 2). Over this limited imaging depth, the resolution integrals calculated from 0–10 mm depth for the Vevo 770 RMV 704 (tracking) and the Vevo 2100 transducers are more than three times greater than those obtained from the clinical scanners and they have the smallest characteristic resolutions. The resolution integral values presented in Table 3 effectively form an order of merit for preclinical imaging, with the Vevo 770 (particularly when used in tracking mode) and Vevo 2100 being the scanners of choice.

## Conclusion

In this study, a quantitative assessment of the abilities of a series of nine ultrasound transducers to image at depths associated with preclinical applications was undertaken. The resolution integral measurements obtained over a 0–10 mm depth range enabled the transducers to be ranked in order of merit for preclinical imaging. These results were consistent with the data acquired during a qualitative *in vivo* assessment to determine the ability of six of the transducers to image follicles within the ovaries of small animals.

This study demonstrates the ability of the resolution integral, implemented using two Edinburgh Pipe Phantoms, to quantify the acoustic characteristics of transducer/scanner combinations for specific imaging applications, in this instance over a 10 mm depth for preclinical applications. Our long-term objective is to use this phantom to routinely assess the imaging performance of preclinical scanners.

## Figures and Tables

**Table 1:** Details of ultrasound scanners and transducers assessed

Manufacturer	Scanner	Transducers	Frequency of operation (MHz)
Dynamic Imaging	Diasus	L8-16	8–16
Dynamic Imaging	Diasus	L10-22	10–22
Philips	iU22	L17-5	5–17
Philips	iU22	L15-7io	7–15
Sonosite	MicroMaxx	HFL38/13-6	6–13
GE	Vivid 5	i13Lv	10
Visualsonics	Vevo 770	RMV 704	20–60
Visualsonics	Vevo 2100	MS550D	22–55
Visualsonics	Vevo 2100	MS550S	32–56

**Table 2:** Resolution integral, characteristic resolution and depth of field measurements for each of the transducers

Scanner	Transducers	Resolution integral, R	Characteristic resolution, D_R_ (μm)	Depth of field, L_R_ (mm)
Vevo 770 (tracking)	RMV 704	72	93	6.7
Vevo 770 (mean, non-tracking)	RMV 704	25	132	3.3
Vevo 2100	MS550S	56	188	10.5
Vevo 2100	MS550D	55	197	10.9
Diasus	L10-22	44	520	23
iU22	L15-7io	72	600	43
Diasus	L8-16	55	600	33
iU22	L17-5	78	630	49
Vivid 5	i13Lv	35	720	25
MicroMaxx	HFL38/13-6	57	840	48

Vevo 770 (mean, non-tracking) was the mean of three sets of measurements taken using the same Edinburgh Pipe Phantom over a period of 15 months. Vevo 770 (tracking) was calculated by tracking the vertical extent of each pipe through the phantom to simulate preclinical use. The results are ranked with respect to characteristic resolution such that those transducers with smallest characteristic resolution are at the top of the table.

**Table 3:** Resolution integral, characteristic resolution and depth of field for each of the transducers measured over a 0–10 mm depth range

Scanner	Transducers	Resolution integral, R	Characteristic resolution, D_R_ (μm)	Depth of field, L_R_ (mm)
Vevo 770 (tracking)	RMV 704	72	93	6.7
Vevo 770 (mean, non-tracking)	RMV 704	25	132	3.3
Vevo 2100	MS550S	51	181	9.2
Vevo 2100	MS550D	47	181	8.5
Diasus	L8-16	17	480	8.2
Diasus	L10-22	15	490	7.5
iU22	L15-7io	16	530	8.5
Vivid 5	i13Lv	15	560	8.3
iU22	L17-5	12	600	7.1
MicroMaxx	HFL38/13-6	7	930	6.8

The results are ranked with respect to characteristic resolution such that those transducers with smallest characteristic resolution are at the top of the table.

**Table 4:** Qualitative *in vivo* assessment of six transducers, ranked according to their ability to detect follicles within marmoset ovaries

Rank	Scanner/transducer combinations
1	Vevo 770 RMV 704
2	Diasus L8-16, Diasus L10-22, Philips iU22 L17-5, Philips iU22 L15-7io
3	MicroMaxx HFL38/13-6

Detection rates were highest with the Vevo 770 RMV 704 probe and lowest with the MicroMaxx probe, compared with the Diasus L8-16, Diasus L10-22, Philips iU22 L17-5 and iU22 L15-7io, the latter four probes giving similar detection rates.
